# Lifespan of long‐lived growth hormone receptor knockout mice was not normalized by housing at 30°C since weaning

**DOI:** 10.1111/acel.13123

**Published:** 2020-02-28

**Authors:** Yimin Fang, Samuel McFadden, Justin Darcy, Erin R. Hascup, Kevin N. Hascup, Andrzej Bartke

**Affiliations:** ^1^ Department of Neurology Southern Illinois University School of Medicine Springfield IL USA; ^2^ Department of Internal Medicine Southern Illinois University School of Medicine Springfield IL USA; ^3^ Department of Pharmacology Southern Illinois University School of Medicine Springfield IL USA; ^4^ Department of Molecular Biology, Microbiology and Biochemistry Southern Illinois University School of Medicine Springfield IL USA; ^5^ Present address: Section on Integrative Physiology and Metabolism Joslin Diabetes Center Harvard Medical School Boston MA USA

**Keywords:** GHRKO, growth hormone receptor, lifespan, metabolism, temperature, thermogenesis

## Abstract

Growth hormone receptor knockout (GHRKO) mice are remarkably long‐lived and have improved glucose homeostasis along with altered energy metabolism which manifests through decreased respiratory quotient (RQ) and increased oxygen consumption (VO_2_). Short‐term exposure of these animals to increased environmental temperature (eT) at 30°C can normalize their VO_2_ and RQ. We hypothesized that increased heat loss in the diminutive GHRKO mice housed at 23°C and the consequent metabolic adjustments to meet the increased energy demand for thermogenesis may promote extension of longevity, and preventing these adjustments by chronic exposure to increased eT will reduce or eliminate their longevity advantage. To test these hypotheses, GHRKO mice were housed at increased eT (30°C) since weaning. Here, we report that contrasting with the effects of short‐term exposure of adult GHRKO mice to 30°C, transferring juvenile GHRKO mice to chronic housing at 30°C did not normalize the examined parameters of energy metabolism and glucose homeostasis. Moreover, despite decreased expression levels of thermogenic genes in brown adipose tissue (BAT) and elevated core body temperature, the lifespan of male GHRKO mice was not reduced, while the lifespan of female GHRKO mice was increased, along with improved glucose homeostasis. The results indicate that GHRKO mice have intrinsic features that help maintain their delayed, healthy aging, and extended longevity at both 23°C and 30°C.

## Graphical abstract

Chronic housing at 30°C of long‐lived growth hormone receptor knockout (GHRKO) mice since weaning did normalize the examined parameters of energy metabolism and glucose homeostasis. The lifespan of male GHRKO mice was not reduced, while the lifespan of female GHRKO mice was increased, along with improved glucose homeostasis. The results indicate that GHRKO mice have intrinsic features that help maintain their delayed, healthy aging, and extended longevity at both 23°C and 30°C.

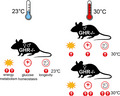

## 1 INTRODUCTION

Environmental temperature (eT) affects metabolic rate and is a key determinant of aging rate and longevity in exothermic (aka poikilothermic or “cold‐blooded”) animals (Keil, Cummings, & de Magalhaes, 2015). In endothermic (aka homeothermic or “warm‐blooded”) animals, the energy expenditure required to maintain core body temperature is the lowest at thermoneutrality, which differs among species (Lodhi & Semenkovich, 2009). For mice, thermoneutrality is approximately 30–32°C (Lodhi & Semenkovich, 2009), while for humans, it is 25–30°C (Kingma, Frijns, Schellen, & van Marken Lichtenbelt, 2014). Consequently, at eT in which humans wearing light clothing feel comfortable (20–23°C), mice experience chronic, mild cold stress and over one‐third of their energy expenditure is devoted to maintaining their core body temperature (Reitman, 2018). This demand for thermogenesis greatly impacts their metabolism, and it has been suggested that mice studied at eT higher than standard animal room temperature of 20–23°C or at thermoneutrality (30–32°C) may be a better model for human energy homeostasis (Keijer, Li, & Speakman, 2019). Indeed, a recent study demonstrating that brown adipose tissue (BAT) from mice housed at thermoneutrality is more similar to that of humans, and therefore has more translatability, has gained considerable attention in the metabolism field (de Jong et al., 2019). However, deciding what eT is the most appropriate for results obtained in mice to be translatable to humans continues to be debated (Fischer, Cannon, & Nedergaard, 2019; Ganeshan & Chawla, 2017; Keijer et al., 2019).

Housing mice at thermoneutral temperature affects their energy expenditure (Cannon & Nedergaard, 2011), food intake (Fregly, Marshall, & Mayer, 1957), body composition (Cui et al., 2016), glucose metabolism (Rippe, Berger, Boiers, Ricquier, & Erlanson‐Albertsson, 2000), cancer risk (Hylander & Repasky, 2016), and beneficial effects of calorie restriction (Koizumi et al., 1996; Overton & Williams, 2004). Although the influence of metabolic rate on longevity has been studied and debated for decades, the cause and effect relationships among different aspects of mitochondrial function, thermogenesis, and energy metabolism and the process of aging are poorly understood and controversial (Manini, 2010; Sohal, Mockett, & Orr, 2002).

Mice with genetic deficiencies in somatotropic signaling, including the levels (Manini, 2010) and actions of growth hormone (GH) (Masternak & Bartke, 2012), have a major longevity advantage over genetically normal (“wild‐type”) animals (Bartke, 2017). Hypopituitary Ames dwarf (Prop1^df^) mice with deficiency of GH, thyroid‐stimulating hormone (TSH), and prolactin (Bartke & Brown‐Borg, 2004), and GH receptor knockout (GHRKO) mice with GH resistance (Zhou et al., 1997), are remarkably long‐lived and exhibit many features of the delayed, healthy aging, and extended longevity (Bartke & Brown‐Borg, 2004; Basu, Qian, & Kopchick, 2018). Many phenotypic characteristics shared by these mutants are believed to represent mechanisms of extended longevity. These characteristics include improved insulin sensitivity and glucose clearance (Bartke, 2017), altered energy metabolism manifested by increased mass and activity of BAT (Darcy et al., 2018; Li, Knapp, & Kopchick, 2003), decreased respiratory quotient (RQ) and increased oxygen consumption (VO_2_) per unit of body mass and heat production (Westbrook, Bonkowski, Strader, & Bartke, 2009), which, surprisingly, are associated with reduced body temperature (Hauck, Hunter, Danilovich, Kopchick, & Bartke, 2001; Hunter, Croson, Bartke, Gentry, & Meliska, 1999).

Based on these observations, we hypothesized that increased heat loss and the consequent increase in energy demand for thermogenesis in Ames dwarf and GHRKO mice housed at standard animal room temperature, which induce alterations in energy metabolism (including increased VO_2_ and reduced RQ), may contribute to their delayed, healthy aging, and extended longevity. To test this hypothesis, we first examined whether alterations in energy metabolism would be reduced or eliminated when these diminutive mutants were housed at or near thermoneutral temperature. Indeed, the increase in VO_2_ and the reduction in RQ in Ames dwarf and GHRKO mice disappeared or were severely attenuated after 24 hr of acclimation to temperature of 30°C (Westbrook, 2012). Exposing adult male Ames dwarf mice to 30°C for 4 months led to the normalization of both VO_2_ and RQ, as well as a partial normalization (i.e., impairment) of glucose homeostasis (Darcy et al., 2018). From these findings, we further hypothesized that chronic exposure of these mutants to increased eT (30°C) might reduce, or eliminate, their longevity advantage. However, we did not know to what extent findings obtained in hypopituitary and hypothyroid Ames dwarf mice may predict responses to chronically elevated eT in GHRKO mice with isolated GH resistance. Thus, GHRKO mice were exposed to the increased eT (30°C) starting at the age of 3 weeks (immediately postweaning). We sought to determine whether characteristics associated with delayed aging in GHRKO mice are normalized by extended (months to years) exposure to 30°C and to compare their metabolic responses to those of sex‐ and age‐matched mice housed at standard animal room temperature (23°C).

Here, we report that contrasting with the effects of short‐term exposure of adult GHRKO mice to 30°C, transferring juvenile GHRKO mice to chronic housing at 30°C did not normalize the examined parameters of energy metabolism and glucose homeostasis. Moreover, despite a drastic decrease in the expression levels of thermogenesis genes in interscapular BAT (iBAT), core body temperature was elevated in response to increased eT, the lifespan of male GHRKO mice was not reduced, and the lifespan of female GHRKO mice was actually increased along with improved glucose homeostasis.

## 2 RESULTS

### 2.1 Body composition of GHRKO mice was altered after chronically housing at 30°C

**Figure 1:**
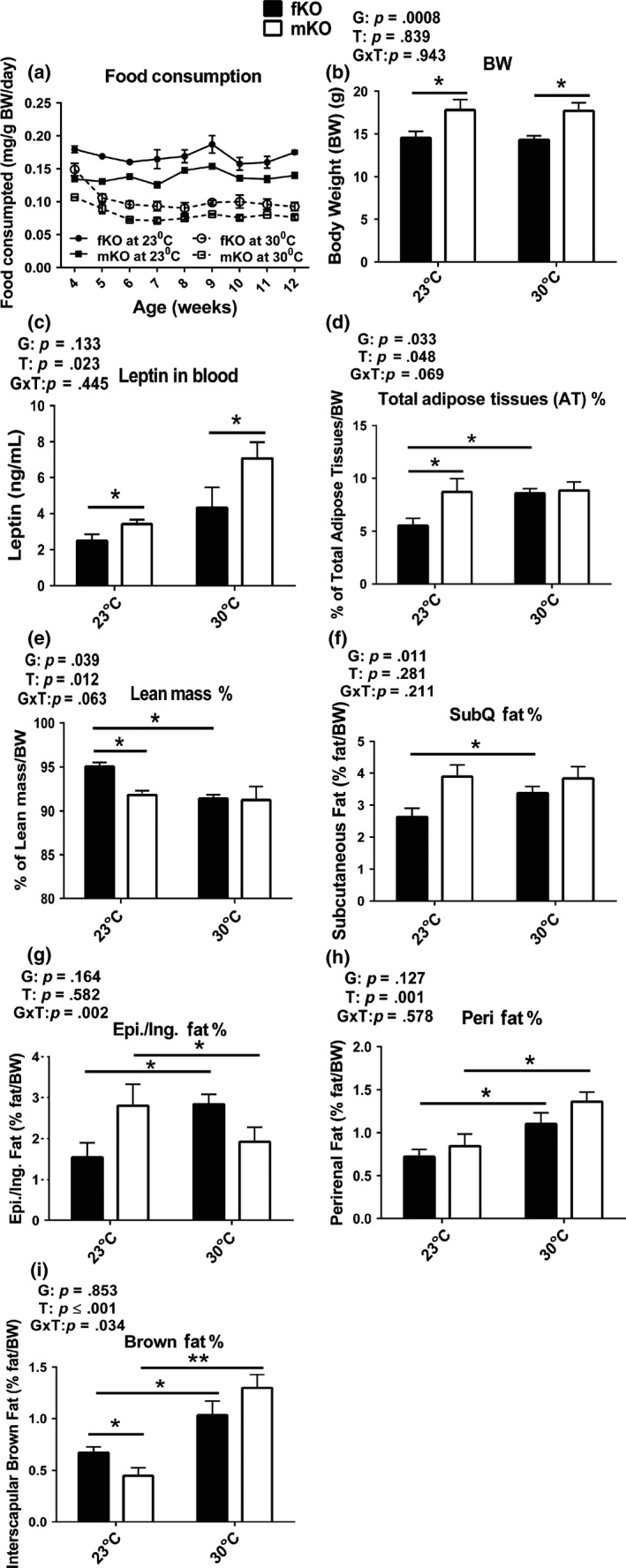
Body weight of GHRKO mice chronically housed at increased eT (30°C) did not change and body composition was altered in sex‐dependent manner. (a) Food consumption: starting measurement at the first week postweaning for 9 weeks. *n* = 3 cages for each group (fKO, females; mKO, males). (b) Body weight (BW). (c) Plasma leptin levels. (d‐i) Body composition (g: Epi. denotes epididymal fat depot in the males and Ing. denotes inguinal fat depot in the females). Body weight and composition, and leptin levels were measured at 21 months of age. Data are means ± *SEM* (*n* = 8–9). G = gender, T = temperature, G × T = interaction. An asterisk (*) indicates *p* ≤ .05, two asterisks (**) indicate *p* ≤ .01 by two‐tailed Student's *t* test

GHRKO mice were housed at 30°C starting at the age of 3 weeks, monitored for changes in glucose homeostasis and energy metabolism at 13 months of age (after approximately 1 year at increased eT), and sacrificed at the age of 21 months to collect blood and tissues for analyses. Food consumption was determined starting the first week after housing at 30°C. The results showed that although female GHRKO mice had higher food consumption per gram body weight than male mice (Figure 1a), their body weight (Figure 1b) and circulating leptin levels (Figure 1c) were lower than in the males at both eT conditions (23°C or 30°C). Interestingly, body weight of either the females or the males did not differ between animals housed at 23°C and 30°C (Figure 1b). However, body composition in the females was altered by chronic housing at 30°C, reflected by the observation that the percentage of total adipose tissues (AT) increased (Figure 1d) and of total lean mass decreased (Figure 1e) compared to the females housed at 23°C. These two parameters of body composition in the males did not change when chronically housed at 30°C (Figure 1d,e). Moreover, each fat depot was differentially altered in a sex‐dependent pattern. At 30°C, the relative mass of each of the four examined fat depots increased in the females (Figure 1f‐i). In the males, the relative mass of perirenal and interscapular brown fat depots increased (Figure 1h,i), while the epididymal fat depot drastically decreased (Figure 1g), and the subcutaneous fat depot did not change (Figure 1f). Thus, chronic housing at 30°C led to an increase in each of the examined fat depots in the females, but not in the males. Collectively, the data indicate that body weight of GHRKO mice chronically housed at increased eT (30°C) did not change and body composition was altered in sex‐dependent manner.

### 2.2 Both male and female GHRKO mice maintained glucose homeostasis and the females further improved insulin sensitivity at 30°C

**Figure 2:**
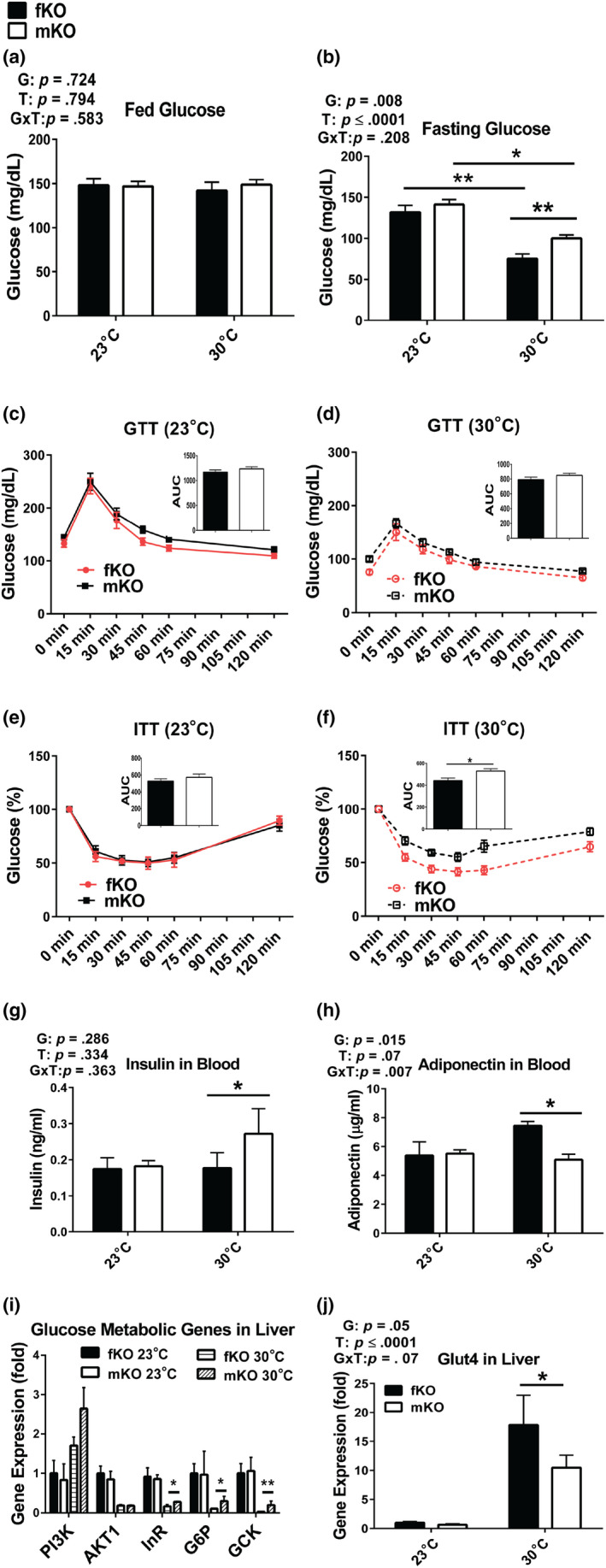
Glucose homeostasis was maintained in male GHRKO mice (mKO) and further improved in female GHRKO mice (fKO) chronically housed at 30°C. (a) Fed glucose levels. (b) Fasting glucose levels. (c‐d) Glucose tolerance test (GTT) at 23°C (c) or 30°C (d) (insertions are area under curve [AUC]). (e‐f) Insulin tolerance test (ITT) at 23°C (e) or 30°C (f) (insertions are area under curve [AUC]). (g) Plasma insulin levels. (h) Plasma adiponectin levels. (i‐j) Gene expression levels in the liver measured by RT–PCR. GTT and ITT were conducted at 13 months of age (after approximately 1 year at increased eT). Insulin and adiponectin levels were measured from plasma and gene expression in the liver collected at sacrifice (21 months of age). Data are means ± *SEM* (*n* = 5–10). G = gender; T = temperature; G × T = interaction. An asterisk (*) indicates *p* ≤ .05; two asterisks (**) indicate *p* ≤ .01 by two‐tailed Student's *t* test

GHRKO mice are GH‐resistant, small, obese, hypoinsulinemic, and highly insulin sensitive. We have previously reported that at 23°C, GHRKO mice had improved glucose metabolism indicated by lower fasting glucose, improved glucose clearance (measured by glucose tolerance test [GTT]), and insulin sensitivity (measured by insulin tolerance test [ITT]) compared to wild‐type mice (Bonkowski et al., 2009; Bonkowski, Rocha, Masternak, Al Regaiey, & Bartke, 2006; Fang et al., 2018). Here, glucose homeostasis was examined at 13 months of age after housing at 30°C for approximately 1 year postweaning. The data showed that although fed glucose levels in both the females and the males did not differ between animals housed at 23°C or 30°C (Figure 2a), fasting glucose levels in both sexes were lower at 30°C than at 23°C. Moreover, at 30°C, fasting glucose levels were more pronouncedly reduced in the females than in the males (Figure 2b). At 23°C, glucose and insulin tolerance in the males were similar to the values measured in the females (Figure 2c,e). At 30°C, glucose tolerance was not different between the males and females (Figure 2d), while the females had higher insulin sensitivity than the males (Figure 2f). Since low insulin levels and increased levels of adiponectin (an enhancer of insulin sensitivity) have been associated with improved glucose metabolism in GHRKO mice (Bartke, 2017; List et al., 2011), we measured insulin and adiponectin levels in the present study. In the females, insulin levels did not differ between animals housed at 23°C and 30°C, while in the males, insulin levels were higher at 30°C than at 23°C (Figure 2g). Adiponectin levels in the males were similar at 23°C and 30°C, while in the females, the levels of adiponectin were higher at 30°C than at 23°C and also higher than the levels measured in the males at either 23°C or 30°C (Figure 2h). The hepatic expression of glucose metabolism‐related genes indicated that at 30°C, the females had relative lower glucose metabolic activity (Figure 2i), but much higher expression of glucose transporter Glut4 than the males (Figure 2j). Together, the results showed that at 30°C, glucose homeostasis was maintained in the males, while it was further improved in the females, possibly associated with increased adiponectin levels and Glut4‐mediated glucose transport.

### 2.3 GHRKO mice maintained relatively high energy metabolism despite reduced uncoupling protein one (UCP1) expression in iBAT

**Figure 3:**
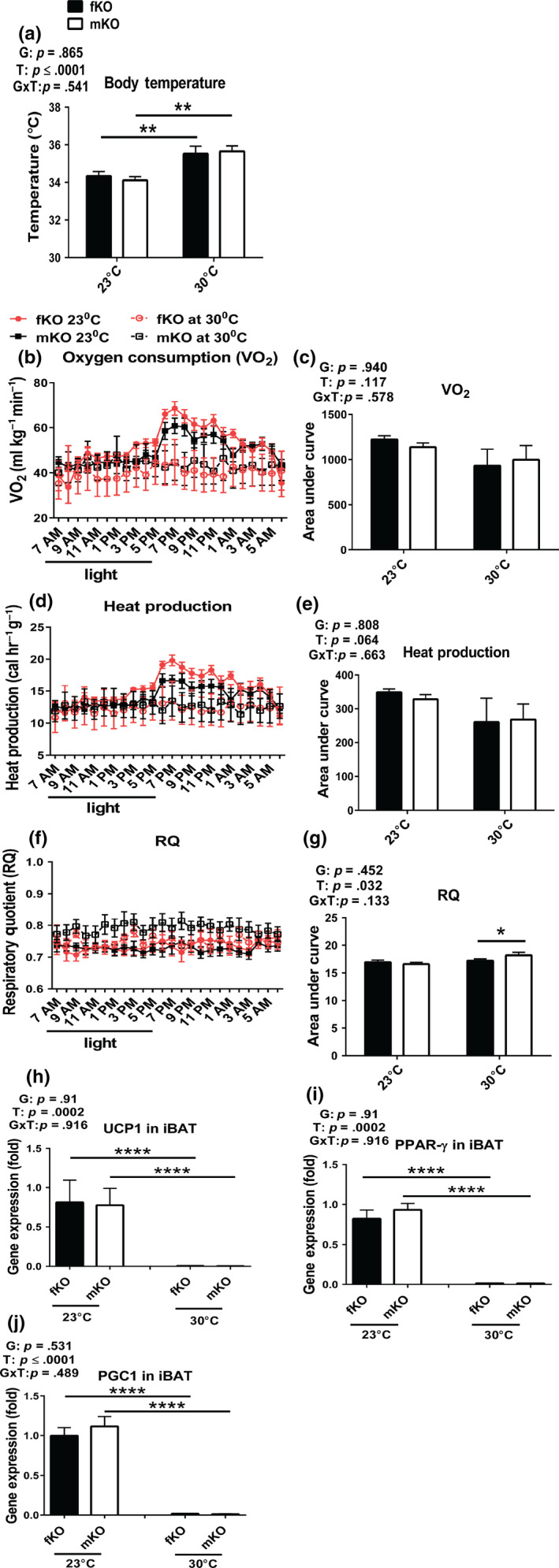
Energy metabolism of GHRKO mice chronically housed at 30°C remained relatively high despite reduced expression of key thermogenic genes in iBAT (fKO, females; mKO, males). (a) Body temperature measured by rectal thermometer at 13 months of age. (b‐c) Oxygen consumption (VO_2_). (d‐e) Heat production. (f‐g) Respiratory quotient (RQ = CO_2_/O_2_). Energy metabolism was measured at 13 months of age. (h‐j) Thermogenic gene expression from iBAT collected at sacrifice (21 months of age). Data are means ± *SEM* (*n* = 5–10). G = gender; T = temperature; G × T = interaction. An asterisk (*) indicates *p* ≤ .05; two asterisks (**) indicate *p* ≤ .01, four asterisks (****) indicate *p* ≤ .0001 by two‐tailed Student's *t* test

At standard room temperature (23°C), GHRKO mice have improved energy metabolism indicated by increased VO_2_ and decreased RQ (Westbrook et al., 2009). They also have an increase in the relative weight of iBAT and elevated expression of UCP1 (both mRNA and protein levels) in iBAT (Li et al., 2003), the key site of nonshivering thermogenesis (Cannon & Nedergaard, 2011). These parameters of energy metabolism of GHRKO mice were considered to be driven by high energy demand to compensate for heat loss at 23°C due to their increased surface area to mass ratio. When the mice were chronically housed at 30°C, presumably their increased thermogenic demand would have been diminished or eliminated. Thus, we hypothesized that their metabolic characteristics will be normalized. The results from the current study showed that GHRKO mice chronically housed at 30°C had higher body temperature than the animals housed at standard room temperature (23°C) (Figure 3a), while their VO_2_ (Figure 3b,c) and heat production (Figure 3d,e) had decreased (although the decrease did not reach statistical significance). This was associated with loss of the pronounced diurnal rhythm of VO_2_ and heat production (Figure 3b,d). The peaks of VO_2_ (Figure 3b) and heat production (Figure 3d), measured at 23°C soon after lights off, were no longer evident at 30°C. Respiratory quotient in the females did not change in response to increased eT, while it was increased in the males at 30°C, indicating a shift in metabolic substrate utilization from fat (RQ ~ 0.7) to more carbohydrates (RQ for mixed fat and carbohydrates ~ 0.8) (Prentice et al., 2013) (Figure 3f,g).

UCP1 is predominantly expressed in BAT and serves to uncouple the electron transport chain from ATP production (Busiello, Savarese, & Lombardi, 2015) and thus mediates nonshivering thermogenesis (Bargut, Aguila, & Mandarim‐de‐Lacerda, 2016). In the current study, to explore whether the reduction in energy metabolism at increased eT reflected suppression of UCP1‐mediated nonshivering thermogenesis, the expression of UCP1, along with two of its main transcriptional regulators (PPAR‐γ and PGC1α) in iBAT were examined. The data demonstrated that the expression levels of these genes in iBAT were decreased to almost undetectable levels in both the females and males exposed to 30°C (Figure 3h‐j). Presumably, at 30°C, the UCP1‐dependent thermogenesis in iBAT of GHRKO mice was almost abolished, and other thermogenesis, such as the UCP1‐independent nonadaptive and basal thermogenesis (Feldmann, Golozoubova, Cannon, & Nedergaard, 2009), must have been responsible for the majority of thermogenesis. This was indicated by the relatively modest (25%–30%) reduction of VO_2_ (Figure 3b,c) and heat production (Figure 3d,e).

### 2.4 Lifespan of GHRKO mice was not reduced by housing at 30°C since weaning

**Figure 4:**
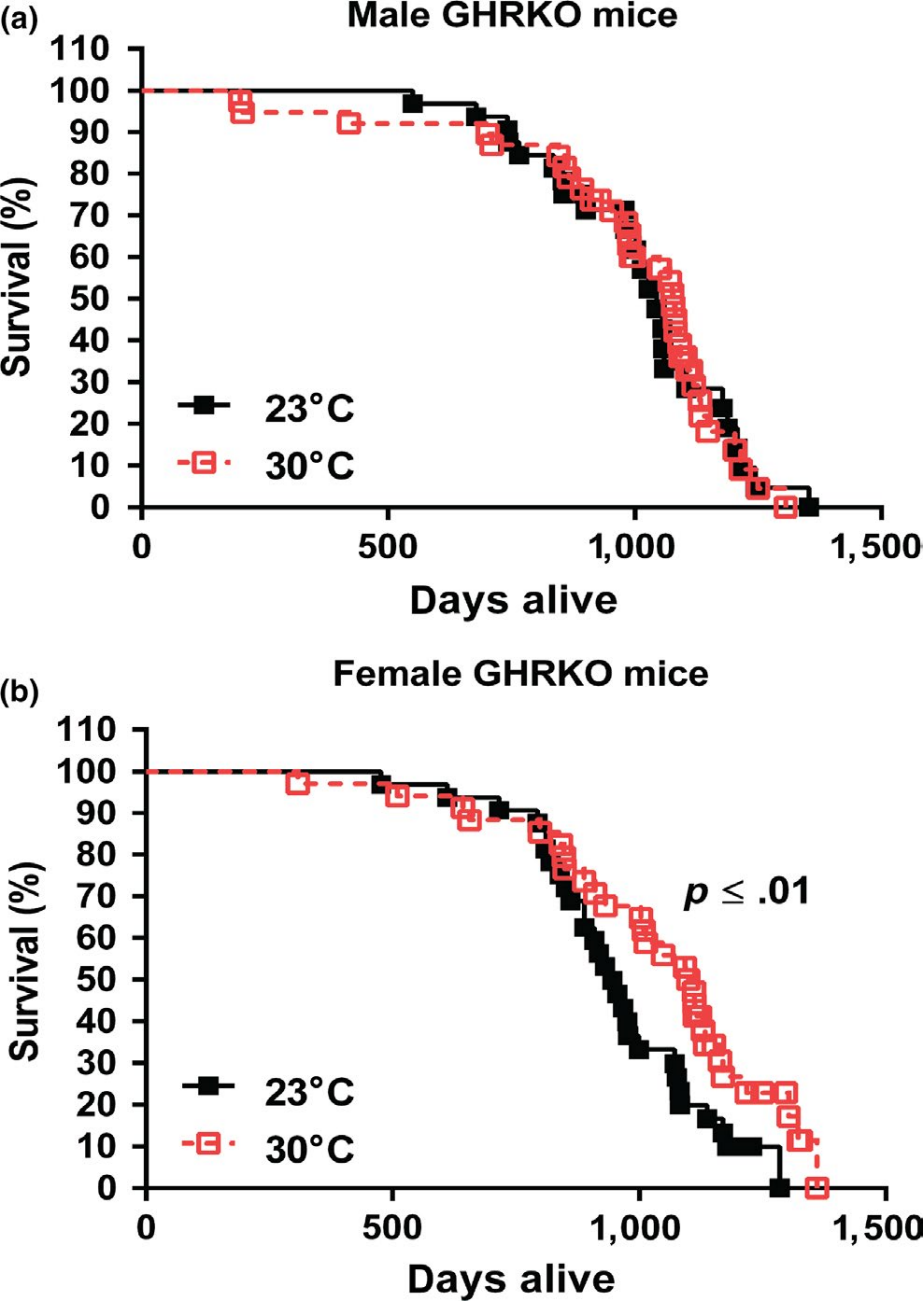
Lifespan of GHRKO mice chronically housed at 30°C was not changed in the males and further extended in the females. (a) Survival curve for male GHRKO mice at 23°C (*n* = 32) and 30°C (*n* = 35). (b) Survival curve for female GHRKO mice at 23°C (*n* = 35) and 30°C (*n* = 34). A separate cohort was used for the longevity study without any interventions until natural end of life. Data represented as mean ± *SEM*. *p* values for lifespan were calculated by a log‐rank test followed by a Tukey post hoc analysis

The results described above suggested that GHRKO mice chronically housed at 30°C maintained their glucose homeostasis and relatively high energy metabolism, the major features associated with the delayed, healthy aging and extended longevity of these animals. Whether GHRKO mice would still have extended lifespan at 30°C remained to be determined. To answer this question, we conducted a longevity experiment to test our hypothesis that chronic exposure of GHRKO to increased eT (30°C) might reduce, or eliminate, their longevity advantage. The longevity study was conducted by housing a separate cohort of both female and male GHRKO mice at 30°C or 23°C since weaning. Contrary to our hypothesis, the longevity data showed that the lifespan of male GHRKO mice did not change at 30°C compared to the males housed at 23°C (Figure 4a), and the survival curve of the males housed at 30°C almost completely overlapped the curve of the males housed at 23°C (Figure 4a). Interestingly, at 30°C, instead of being decreased, the lifespan of female GHRKO mice was further extended (Figure 4b). The lifespan of the females housed at 30°C was increased by 14% compared to the females housed at 23°C (*p* ≤ .01) (Table 1 and S1, S2). In addition, comparison of the age of the longest surviving 20% of animals in each group indicated that maximum longevity of GHRKO females was also increased at 30°C (Table 1 and S1, S2).

**Table 1:** Lifespan for GHRKO mice housed at 23°C or 30°C since weaning

Gender	Temperature	*n*	Lifespan (days)	Maximum lifespan (days)
Female	23°C (control)	35	970 ± 39	1,183 ± 24
	30°C	34	1,104 ± 41**	1,296 ± 18**
Male	23°C (control)	32	1,028 ± 45	1,197 ± 28
	30°C	35	1,077 ± 40	1,212 ± 24

Note

Data represented as mean ± *SEM*. *p* values for lifespan were calculated by a log‐rank test followed by a Tukey post hoc analysis, and maximum lifespan was estimated by calculating average longevity of the oldest surviving 20% of animals in each group and comparing them using conditional Student's *t* tests between the group at 23°C and the group at 30°C. Two asterisks (**) indicate *p* ≤ .01.

## 3 CONCLUSIONS AND DISCUSSION

Results of this study indicate that the remarkable extension of longevity is a robust, intrinsic characteristic of GHRKO mice, and that it persists in animals maintained at increased eT (30°C) since weaning. Average longevity of GHRKO mice housed at 23°C in the current study resembled the values measured in the previous studies (Bonkowski et al., 2006; Coschigano, Clemmons, Bellush, & Kopchick, 2000; List et al., 2011). Similarly, longevity of WT mice from the same strain housed at 23°C appears to resemble the values recorded in the previous studies (Bonkowski et al., 2006; Fang et al., 2018). Interestingly, data available to date suggest that chronic housing at 30°C has no, or very little, impact on the longevity of WT mice from this genetically heterogeneous strain. Koizumi et al. (1996) reported that housing at 30°C did not significantly affect longevity of autoimmune‐prone MRL mice, but reduced longevity of C57BL/6 mice subjected to energy restriction (ER) by diminishing the protective impact of ER and ER‐induced torpor on development of lymphoma (Koizumi et al., 1996). GHRKO mice chronically housed at 30°C had higher body temperature than the animals housed at standard room temperature (23°C) (Figure 3a), but still appeared to maintain somewhat lower body temperature than the control WT mice at either 23°C or 30°C. This is consistent with the observation that lowering core body temperature extended lifespan independent of CR (Conti et al., 2006). In GHRKO mice housed at 30°C, parameters of energy metabolism associated with thermogenesis (VO_2_ and heat production) lost their diurnal rhythm (Figure 3b,d). Moreover, the expression of key thermogenic genes in iBAT, including UCP1, was decreased to almost undetectable levels (Figure 3h‐j). These responses to increased eT likely reflected the suppression of UCP1‐mediated nonshivering thermogenesis in iBAT, the key site of nonshivering thermogenesis (Cannon & Nedergaard, 2011). However, this reduction of thermogenesis apparently accounted for only a small proportion of total energy metabolism (Figure 3c,e), since VO_2_ (Figure 3b) and heat production (Figure 3d) during the light period and late at night were not distinguishable in animals housed at 30°C from those housed at 23°C. At 30°C, the advantageous characteristic of low RQ (indicating preference for fat usage as energy substrate) was not altered in the females, and in the males, it remained diminished, but still in the range of mixed fat and carbohydrates usage (Figure 3f,g). Additionally, glucose clearance and insulin sensitivity, the major physiological parameters of glucose homeostasis, were not altered in the males, while the insulin sensitivity was further improved in the females chronically housed at 30°C. Together, this invites speculation that these longevity‐associated characteristics contributed to the maintenance of the extended longevity in the males and to the further extension of longevity in the females at 30°C as compared to 23°C (Table 1, s1, s2 and Figure 4).

Thermogenesis can be separated into three components (Feldmann et al., 2009): basal metabolism (BMR: resting metabolic rate), UCP1‐independent nonadaptive thermogenesis, and UCP1‐dependent adaptive thermogenesis. In the current study, we cannot directly define functions of UCP1 in iBAT. However, our data indicate that the high energy metabolism, which is a characteristic of GHRKO mice (Westbrook et al., 2009), presumably includes a small proportion of energy expenditure from UCP1‐dependent thermogenesis. In support of this interpretation, UCP1 expression in iBAT at 30°C was decreased to almost undetectable levels (Figure 3h), which was associated with only a modest decline in overall energy expenditure (Figure 3c,e). Therefore, the basal and the UCP1‐independent thermogenesis must be relatively high, accounting for 70%–75% of VO_2_ (Figure 3b,c) and heat production (Figure 3d,e). These findings also indicate that UCP1‐mediated nonshivering thermogenesis in iBAT of GHRKO mice housed at 30°C might not be a major contributor of their elevated body temperature (Figure 3a). It is plausible that existence of other UCP1‐independent thermogenesis, in both adipocytes and muscle, such as creatine and calcium cycling (Betz & Enerback, 2018), might be responsible for the elevation of body temperature of GHRKO mice housed at 30°C. This leads to the speculation that UCP1‐independent energy metabolism is negatively regulated by somatotropic signaling, which is deficient in GHRKO mice. This warrants future investigation.

The higher energy metabolism at 30°C could also contribute to maintenance (in the males) or improvement (in the females) of glucose homeostasis in GHRKO mice (Figure 2d,f). Coexistence of increased adiposity with improved insulin sensitivity is one of the characteristics of these, and other GH signaling‐deleted long‐lived mutants (Bartke, 2017). In GHRKO mice, visceral adipose tissues appear to be unique in that they were previously shown to promote insulin sensitivity rather than insulin resistance (Bennis et al., 2017; Masternak et al., 2012). The improved glucose homeostasis in the females housed at 30°C could be explained by the improvement in insulin sensitivity (Figure 2f) in spite of increased adiposity (Figure 1d) and is likely due to increased adiponectin secretion (Figure 2h), as well as higher glucose transporting activity (Figure 2j).

Data from the current study failed to support our original hypothesis that chronic exposure of these diminutive mutants to increased eT will reduce or eliminate their longevity advantage, by preventing increased heat loss and the consequent increase in energy demand for thermogenesis at standard animal room temperature of 23°C. In contrast to our previous studies in adult mice (Darcy et al., 2018; Westbrook et al., 2009), juvenile mice in the current study might adapt to the eT. Thus, the differences we observed in the current study are not of the same magnitude as what was observed in these mice and Ames dwarfs exposed to 30°C for 24 hr in our previous studies (Westbrook, 2012), or in adult Ames dwarfs housed at this eT for 4 months (Darcy et al., 2018). We do not currently understand the precise mechanism for this discrepancy, but believe that the most likely explanation is the difference in the age of the animals at the time of transferring them from 23°C to 30°C. The suggested importance of exposing juvenile versus adult mice to increased eT is consistent with the well‐documented potential of early life nutritional, hormonal, or environmental interventions to influence adult phenotypic characteristics, including rate of aging and longevity (Barker, 1997; Bartke & Quainoo, 2018; Sun et al.,^2017^). The data generated in adult Ames dwarf mice concerning energy metabolism (Darcy et al., 2018) are likely influenced by profound suppression of thyroid function in these animals.

The data from the current study demonstrate that the UCP1‐dependent thermogenesis in iBAT of GHRKO mice might be the major contributor to compensate for extra heat loss by driving high energy metabolism at standard room temperature (23°C), which was eliminated or minimized at 30°C. However, GHRKO mice exposed to 30°C since weaning still maintained their high metabolism even though UCP1‐dependent thermogenesis (energy metabolism) in iBAT was severely attenuated. The remaining forms of thermogenesis (energy metabolism) in GHRKO mice at 30°C are the most likely crucial driving forces to maintain glucose homeostasis in these mice. This could explain why the lifespan of GHRKO mice was not normalized (shortened) by the increased eT (30°C).

The current study provides some insight into the mechanistic relations of the increased heat loss and the consequent increase in energy demand for thermogenesis at standard room temperature (23°C) to the delayed, healthy aging and extended longevity in GHRKO mice. GHRKO mice housed at increased eT (30°C) had increased body temperature presumably without UCP1‐mediated nonshivering thermogenesis in iBAT and had 25%–30% reduction of energy metabolism compared to the mice housed at 23°C (Figure 3). However, the mice still remained high metabolic activity, maintained, or improved glucose metabolism, and lifespan was not normalized. The results implicate that GHRKO mice have intrinsic characteristics that help maintain homeostasis at both 23°C and 30°C. It remains to be elucidated how intrinsic high energy and glucose metabolism are regulated and how they might explain extended longevity of these animals.

## 4 EXPERIMENTAL PROCEDURES

### 4.1 Mice maintenance and longevity study

All animal procedures were approved by the Laboratory Animal Care and Use Committee of Southern Illinois University School of Medicine. Mice were group‐housed (five animals per cage) under temperature‐ and light‐controlled conditions (23°C/30°C ± 1°C, 12‐hr light/12‐hr dark cycle) with ad libitum access to food (Chow 5001 with 23.4% protein, 5% fat, and 5.8% crude fiber; LabDiet PMI Feeds) and water. Our breeding colony was developed by crossing male GHRKO mice with 129 Ola/BALB/c background generated in Dr. John J. Kopchick's laboratory (Coschigano et al., 2000) with female normal mice derived from crossing of C57BL/6 and C3H strains, and thereafter breeding of the resulting animals in a closed colony without brother × sister mating. Thus, the animals have a heterogeneous genetic background. Starting at 3 weeks of age immediately postweaning, GHRKO mice were housed at 23°C or 30°C. A group of animals were sacrificed after exposure to 23°C or 30°C eT for approximately 20 months. For the longevity study, animals remained at 23°C or 30°C eT until natural end of life. Animals that appeared near death were euthanized, and date of euthanasia was considered date of death.

### 4.2 Glucose tolerance test (GTT) and insulin tolerance test (ITT)

GTT or ITT was carried out as described previously (Fang et al., 2018). Sixteen‐hour‐fasted mice underwent GTT by intraperitoneal (i.p.) injection with 1 g glucose per kg of body weight (BW). Blood glucose levels were measured at 0, 15, 30, 45, 60, and 120 min with a PRESTO glucometer (AgaMatrix). For ITT, nonfasted mice were injected i.p. with 1 IU porcine insulin (Sigma) per kg of BW. Blood glucose levels were measured at 0, 15, 30, 45, 60, and 120 min. The data for GTT are presented as absolute value, and for ITT are presented as a percentage of baseline glucose.

### 4.3 Indirect calorimetry

Indirect calorimetry was performed as previously described (Fang et al., 2018) using AccuScan Metabolic System (AccuScan Instruments). In this system, mice are housed individually in metabolic chambers with ad libitum access to food (Chow 5001; LabDiet PMI Feeds) and water. After a 24‐hr acclimation period, VO_2_ and RQ measurements were collected every 10 min. All comparisons are based on animals studied simultaneously in eight different chambers connected to the same O_2_, CO_2_, and light beam sensors. Gas samples were collected and analyzed every 10 min per animal, and the data were averaged for each hour.

### 4.4 Assessment of blood chemistry

Plasma was collected from animals anesthetized with isoflurane by cardiac puncture at sacrifice. The blood was mixed with EDTA, followed by centrifugation at 10,000 *g* for 15 min at 4°C for plasma collection. Per the manufacturer's protocol, insulin or leptin was measured with a Mouse Insulin ELISA Kit or Mouse Leptin ELISA Kit (Crystal Chem), adiponectin with a Mouse Adiponectin ELISA Kit (Linco Research).

### 4.5 RT–PCR

mRNA expression was analyzed by quantitative RT–PCR as previously described (Fang et al., 2018) using the StepOne System and SYBR Green MasterMix (Applied Biosystems). RNA was extracted using an RNeasy mini kit or RNeasy Lipid Tissue Mini Kit (Qiagen) following the manufacturer's instructions. Relative expression was calculated as previously described (Masternak et al., 2012).

### 4.6 Statistical analysis

Statistical analyses were conducted using a two‐way ANOVA to test for significance of sex (males × females), temperature (23°C × 30°C), and an interaction between sex and temperature. Differences between two groups were assessed with unpaired two‐tailed Student's *t* tests. The area under the curve was calculated using the two‐way ANOVA. A log‐rank test followed by a Tukey post hoc analysis between animals at eT of 23°C and 30°C was used for the longevity study. Maximum lifespan was estimated by calculating average longevity of the oldest surviving 20% of animals in each group and comparing them using conditional Student's *t* tests between the group at 23°C and the group at 30°C. Data are presented as means ± *SEM*. All statistical analyses and graphs were completed using Prism 6 (GraphPad Inc, La Jolla, CA, USA).

## CONFLICT OF INTEREST

The authors have no affiliations with or involvement in any organization or entity with any financial interest or nonfinancial interest in the subject matter or materials discussed in this manuscript.

## AUTHOR CONTRIBUTIONS

Y.F. designed and conducted the experiments, analyzed the data, prepared the figures, and wrote the paper. S.M. conducted the experiments and analyzed the data. J.D. helped with writing and conducting the experiments. E.H. and K.H. provided funding and helped with writing the paper. A.B. provided funding, designed experiments, and helped with writing the paper.

## Data Availability

The data that support the findings of this study are available from the corresponding author (YF), upon reasonable request.
