# Supplementary material for: Lifespan of long‐lived growth hormone receptor knockout mice was not normalized by housing at 30°C since weaning
Source: Aging Cell. 2020 Feb 28;19(5):e13123. doi: 10.1111/acel.13123 (PMC7253058; doi:10.1111/acel.13123)
Supplement: Supplementary file 2 [file ACEL-19-e13123-s002.docx]

**Supporting Information listing:**

**Excel workbook** for raw data.

**Table s1**: Data of Figure 4a (lifetime survival for female GHRKO mice at eT 23^0^C or 30^0^C).

| female GHRKO mice at 23^0^C | |  |  |
| --- | --- | --- | --- |
| age-interval (months) | entering age-interval | dead within age-interval | censored within age-interval |
| 5 | 35 | 0 | 0 |
| 6 | 35 | 1 | 0 |
| 7 | 34 | 0 | 0 |
| 8 | 34 | 0 | 0 |
| 9 | 34 | 0 | 0 |
| 10 | 34 | 0 | 0 |
| 11 | 34 | 0 | 0 |
| 12 | 34 | 0 | 0 |
| 13 | 34 | 0 | 0 |
| 14 | 34 | 0 | 0 |
| 15 | 34 | 0 | 0 |
| 16 | 34 | 1 | 0 |
| 17 | 33 | 0 | 0 |
| 18 | 33 | 1 | 0 |
| 19 | 32 | 0 | 0 |
| 20 | 32 | 1 | 0 |
| 21 | 31 | 0 | 0 |
| 22 | 31 | 0 | 0 |
| 23 | 31 | 0 | 0 |
| 24 | 31 | 3 | 0 |
| 25 | 28 | 0 | 0 |
| 26 | 28 | 1 | 0 |
| 27 | 27 | 3 | 0 |
| 28 | 24 | 2 | 0 |
| 29 | 22 | 2 | 0 |
| 30 | 20 | 3 | 0 |
| 31 | 17 | 3 | 0 |
| 32 | 14 | 2 | 0 |
| 33 | 12 | 2 | 0 |
| 34 | 10 | 0 | 0 |
| 35 | 10 | 0 | 0 |
| 36 | 10 | 3 | 0 |
| 37 | 7 | 0 | 0 |
| 38 | 7 | 1 | 0 |
| 39 | 6 | 2 | 0 |
| 40 | 4 | 1 | 0 |
| 41 | 3 | 1 | 0 |
| 42 | 2 | 0 | 0 |
| 43 | 2 | 1 | 0 |
| 44 | 1 | 0 | 0 |

| female GHRKO mice 30^0^C | |  |  |
| --- | --- | --- | --- |
| age-interval (months) | entering age-interval | dead within age-interval | censored within age-interval |
| 9 | 34 | 0 | 0 |
| 10 | 34 | 1 | 0 |
| 11 | 33 | 0 | 0 |
| 12 | 33 | 0 | 0 |
| 13 | 33 | 1 | 0 |
| 14 | 32 | 0 | 0 |
| 15 | 32 | 0 | 0 |
| 16 | 32 | 0 | 0 |
| 17 | 32 | 1 | 0 |
| 18 | 31 | 0 | 0 |
| 19 | 31 | 0 | 0 |
| 20 | 31 | 0 | 0 |
| 21 | 31 | 0 | 0 |
| 22 | 31 | 1 | 0 |
| 23 | 30 | 1 | 0 |
| 24 | 29 | 0 | 0 |
| 25 | 29 | 0 | 0 |
| 26 | 29 | 0 | 0 |
| 27 | 29 | 1 | 0 |
| 28 | 28 | 3 | 0 |
| 29 | 25 | 0 | 0 |
| 30 | 25 | 2 | 0 |
| 31 | 23 | 1 | 0 |
| 32 | 22 | 0 | 0 |
| 33 | 22 | 1 | 0 |
| 34 | 21 | 2 | 0 |
| 35 | 19 | 1 | 0 |
| 36 | 18 | 1 | 0 |
| 37 | 17 | 6 | 0 |
| 38 | 11 | 3 | 0 |
| 39 | 8 | 2 | 0 |
| 40 | 6 | 0 | 0 |
| 41 | 6 | 1 | 0 |
| 42 | 5 | 0 | 0 |
| 43 | 5 | 1 | 0 |
| 44 | 4 | 1 | 0 |
| 45 | 3 | 1 | 0 |
| 46 | 2 | 0 | 0 |

**Table s2**: Data of Figure 4b (lifetime survival for male GHRKO mice at eT 23^0^C or 30^0^C).

| male GHRKO mice at 23^0^C | |  |  |
| --- | --- | --- | --- |
| age-interval (months) | entering age-interval | dead within age-interval | censored within age-interval |
| 16 | 32 | 1 | 0 |
| 17 | 31 | 0 | 0 |
| 18 | 31 | 1 | 0 |
| 19 | 30 | 0 | 0 |
| 20 | 30 | 0 | 0 |
| 21 | 30 | 0 | 0 |
| 22 | 30 | 0 | 0 |
| 23 | 30 | 0 | 0 |
| 24 | 30 | 0 | 0 |
| 25 | 30 | 2 | 0 |
| 26 | 28 | 1 | 0 |
| 27 | 27 | 1 | 0 |
| 28 | 26 | 2 | 0 |
| 29 | 24 | 3 | 0 |
| 30 | 21 | 2 | 0 |
| 31 | 19 | 2 | 0 |
| 32 | 17 | 2 | 0 |
| 33 | 15 | 2 | 0 |
| 34 | 13 | 2 | 0 |
| 35 | 11 | 4 | 0 |
| 36 | 7 | 0 | 0 |
| 37 | 7 | 1 | 0 |
| 38 | 6 | 0 | 0 |
| 39 | 6 | 1 | 0 |
| 40 | 5 | 3 | 0 |
| 41 | 2 | 1 | 0 |
| 42 | 1 | 0 | 0 |
| 43 | 1 | 0 | 0 |
| 44 | 1 | 0 | 0 |
| 45 | 1 | 1 | 0 |
| 46 | 0 | 0 | 0 |

| male GHRKO mice at 30^0^C | |  |  |
| --- | --- | --- | --- |
| age-interval (months) | entering age-interval | dead within age-interval | censored within age-interval |
| 6 | 35 | 0 | 0 |
| 7 | 35 | 2 | 0 |
| 8 | 33 | 0 | 0 |
| 9 | 33 | 0 | 0 |
| 10 | 33 | 0 | 0 |
| 11 | 33 | 0 | 0 |
| 12 | 33 | 0 | 0 |
| 13 | 33 | 0 | 0 |
| 14 | 33 | 1 | 0 |
| 15 | 32 | 0 | 0 |
| 16 | 32 | 0 | 0 |
| 17 | 32 | 0 | 0 |
| 18 | 32 | 0 | 0 |
| 19 | 32 | 0 | 0 |
| 20 | 32 | 0 | 0 |
| 21 | 32 | 0 | 0 |
| 22 | 32 | 0 | 0 |
| 23 | 32 | 1 | 0 |
| 24 | 31 | 1 | 0 |
| 25 | 30 | 0 | 0 |
| 26 | 30 | 0 | 0 |
| 27 | 30 | 0 | 0 |
| 28 | 30 | 1 | 0 |
| 29 | 29 | 2 | 0 |
| 30 | 27 | 2 | 0 |
| 31 | 25 | 0 | 0 |
| 32 | 25 | 1 | 0 |
| 33 | 24 | 4 | 0 |
| 34 | 20 | 0 | 0 |
| 35 | 20 | 1 | 0 |
| 36 | 19 | 7 | 0 |
| 37 | 12 | 2 | 0 |
| 38 | 10 | 3 | 0 |
| 39 | 7 | 0 | 0 |
| 40 | 7 | 2 | 0 |
| 41 | 5 | 0 | 0 |
| 42 | 5 | 1 | 0 |
| 43 | 4 | 0 | 0 |
| 44 | 4 | 1 | 0 |
| 45 | 3 | 0 | 0 |

**Table s3:** Primers for RT-PCR

| **Gene** | **Forward Sequence** | **Reverse Sequence** |
| --- | --- | --- |
| B2M | 5’-AAGTATACTCACGCCACCCA-3’ | 5’-AAGACCAGTCCTTGCTGAAG-3’ |
| PI3K | 5’-GTGCATGGACTGTTTCCAATACA-3’ | 5’-AATGACGGACTTCTCACTTCAC-3’ |
| AKT1 | 5’-ATGAACGACGTAGCCATTGTG-3’ | 5’-TTGTAGCCAATAAAGGTGCCAT |
| Insulin Receptor (InR) | 5’-CTTGGTTATCTTCGAGATGGTCC-3’ | 5’-CCCCACATTCCTCGTTGTCA-3’ |
| Glucose-6-phosphatase (G6P) | 5’-TCTGTCCCGGATCTACCTTG-3’ | 5’-GTAGAATCCAAGCGCGAAAC-3’ |
| Glucokinase (GCK) | 5’- CAACTGGACCAAGGGCTTCAA-3’ | 5’-TGTGGCCACCGTGTCATTC-3’ |
| Uncoupling protein 1 (UCP1) | 5’-AGGCTTCCAGTACCATTAGGT-3’ | 5’-CTGAGTGAGGCAAAGCTGATTT-3’ |
| peroxisome proliferator activated receptor gamma (PPAR-γ) | 5’-ACCCCCTGCTCCAGGAGAT-3’ | 5’-TGCAATCAATAGAAGGAACACGT-3’ |
| PPARG coactivator 1 alpha (PGC1-α) | 5’-GGGTTATCTTGGTTGGCTTTATG-3’ | 5’-TGAACGAGAGCGCATCCTT-3’ |
